# Fabrication, Microstructural Evolution, and Mechanical Properties of SiC/(Hf_0.25_Ta_0.25_Zr_0.25_Nb_0.25_)C/C Nanocomposites

**DOI:** 10.3390/ma17215294

**Published:** 2024-10-31

**Authors:** Zhenyue Wang, Tianci Zhou, Xiantao Yang, Yuenong Liu, Qingbo Wen, Zhaoju Yu

**Affiliations:** 1 Key Laboratory of High Performance Ceramic Fibers, Xiamen University, Xiamen 361005, China; 2 State Key Laboratory of Powder Metallurgy, Central South University, Changsha 410083, China; 3 Xiamen Key Laboratory of Electronic Ceramic Materials and Devices, Xiamen University, Xiamen 361005, China

**Keywords:** single-source precursor, polymer-derived ceramic, spark plasma sintering, sic-based nanocomposites

## Abstract

A dense monolithic SiC/(Hf_0.25_Ta_0.25_Zr_0.25_Nb_0.25_)C/C high-entropy ceramic nanocomposite was prepared using a polymer-derived ceramic (PDC) method combined with spark plasma sintering (SPS). The microstructural evolution and mechanical properties of the obtained nanocomposites were characterized by X-ray diffractometer (XRD), transmission electron microscope (TEM), scanning-electron microscope (SEM), and nanoindentation. The results indicate that the phase composition of SiC/(Hf_0.25_Ta_0.25_Zr_0.25_Nb_0.25_)C/C can be adjusted by modifying the metal content of the single-source precursor (SSP) through molecular design. The resulting precursor exhibits an exceptionally high ceramic yield, with mass retention of over 90% at 1100 °C, which guarantees the densification of the final SiC/(Hf_0.25_Ta_0.25_Zr_0.25_Nb_0.25_)C/C composites. The PDC route facilitates the in situ formation of a high-entropy phase within the ceramic matrix under low temperature pyrolysis conditions. Combined with SPS, a dense monolithic SiC/(Hf_0.25_Ta_0.25_Zr_0.25_Nb_0.25_)C/C nanocomposite was obtained, exhibiting an open porosity of 0.41 vol%, nano-hardness of 27.47 ± 0.46 GPa, elastic modulus of 324.00 ± 13.60 GPa, and fracture toughness of 3.59 ± 0.24 MPa·m^0.5^, demonstrating excellent mechanical properties.

## 1. Introduction

In recent years, the concept of high-entropy has gradually extended from alloys to ceramics. High-entropy ceramics are single-phase solid solutions composed of four or more principal elements with close to equiatomic ratios [1,2]. Multiple principal elements can increase the initial oxidation temperature and peak oxidation temperature of ceramics, or form complex oxides to reduce the oxidation rate of ceramics, effectively improving their intrinsic resistance to oxidation [3,4,5,6].

High-entropy ceramics exhibit characteristics of long-range order in crystal structures and disorder in chemical structures, behaving in four core effects: high-entropy, lattice distortion, sluggish diffusion, and cocktail effect [7]. In high-entropy materials, the atomic radii, valences, and bond lengths and strengths of each principal element differ, resulting in significant lattice distortion within the high-entropy ceramic’s lattice. Harrington et al. [8] prepared high-entropy carbides, such as (Mo_0.2_Nb_0.2_Ta_0.2_V_0.2_W_0.2_)C and (Hf_0.2_Ta_0.2_Ti_0.2_W_0.2_Zr_0.2_)C, which exhibit hardness and modulus values as high as 27 GPa and 533 GPa, 33 GPa and 473 GPa, respectively. The results indicate that lattice distortion structures significantly enhance the hardness of high-entropy ceramics. The complex elemental composition within high-entropy materials acts as an obstacle to atomic diffusion. The sluggish diffusion effect hinders processes such as phase transformation and grain growth, thereby enhancing the material’s oxidation resistance. Although high-entropy ceramics exhibit excellent oxidation and resistance at 2000 °C, issues with susceptibility to oxidation in moderate temperature environments (1200–1600 °C) still persist [9].

To enhance the oxidation resistance of ultra-high temperature ceramics (UHTCs) in moderate- to low-temperature environments, introducing SiC as a second phase to create UHTC/SiC nanocomposites with multiphase is an effective approach [10,11,12,13,14]. In extreme conditions, a dense layer of SiO_2_ can form on the surface of the material or fill the pores generated after the oxidation of monolithic ceramics. This layer inhibits oxygen diffusion within the ceramics, slowing down the oxidation rate and providing effective oxidation protection below 1800 °C [10,11,12,13,14].

Polymer-derived ceramic (PDC) is an advanced method of producing ceramics via crosslinking and pyrolysis of organic polymers. The phase composition as well as microstructure of the PDCs are strongly determined by the molecular structure of the preceramic polymer [15,16]. The PDC technique, known for its polymer-processing capabilities and homogeneous ceramic composition, is considered as ideal method for fabricating complex-shaped or compositionally diverse nanocomposite ceramics [17]. Spark plasma sintering (SPS) is a sintering technique that utilizes pulsed direct current to rapidly heat and consolidate powders [18]. Compared to traditional hot-pressing sintering methods, it offers faster heating rates, lower sintering temperatures, and shorter sintering times, without the need for sintering aids. Moreover, the SPS technology yields smaller ceramic grains, enabling the monolithic material to preserve a refined microstructure [18,19,20]. Combining the advantages of PDC and SPS techniques, we reported the first study on the preparation, microstructural evolution, electrical conductivity, and electromagnetic properties of dense monolithic SiC/HfC_x_N_1−x_/C and SiC/Hf_y_Ta_1−y_C_x_N_1−x_/C nanocomposites [21,22].

The aforementioned preliminary work utilized expensive tetrakis (dimethylamido) hafnium or tetrakis (diethylamido) hafnium as the hafnium source. The presence of nitrogen elements resulted in thermal decomposition of the final ceramics at 1500 °C, leading to thermal weight loss and affecting their high temperature performance [21,23]. Therefore, innovatively, we utilized the highly reactive and cost-effective HfCl_4_ as the hafnium source to synthesize SSPs through the dehydrochlorination reaction with allylhydridopolycarbosilane (AHPCS) [24]. This SSP facilitated the preparation of SiC-HfC-C nanocomposite containing core–shell structures of SiC@C and HfC@C [24]. Further reaction of AHPCS with HfCl_4_ and TaCl_5_ confirmed that the solid solution composition of (Hf_x_Ta_1−x_)C in SiC/(Hf_x_Ta_1−x_)C/C can be controlled by changing the feed ratios of HfCl_4_/TaCl_5_ during synthesis of SSPs. Building upon our previous work, HfCl_4_, TaCl_5_, ZrCl_4_, and NbCl_5_ were introduced into AHPCS to successfully produce a series of liquid SSPs with excellent processability. Through the PDC approach, SiC/(Hf_0.25_Ta_0.25_Zr_0.25_Nb_0.25_)C/C nanocomposites with SiC@C and (Hf_0.25_Ta_0.25_Zr_0.25_Nb_0.25_)C@C core–shell structures were obtained, and the evolution of their phase composition and microstructure during the ceramization process were investigated in detail. Combining PDC and SPS technologies, we fabricated fully dense monolithic SiC/(Hf_0.25_Ta_0.25_Zr_0.25_Nb_0.25_)C/C nanocomposites and investigated the mechanical properties of the prepared ceramic bulk materials.

## 2. Experimental Methods

### 2.1. Materials

HfCl_4_, TaCl_5_, ZrCl_4_, and NbCl_5_ (purity 98%) were provided by Shanghai Macklin Biochemical Technology Co., Ltd (Shanghai, China). CHCl_3_ (AR) was distilled before use. According to the literature [25], AHPCS with the formula [SiH_1.30_(CH3)_0.60_(CH_2_CH=CH_2_)_0.10_CH_2_]_n_ was prepared. Argon and nitrogen gases with a purity of 99.999% were supplied by Linde Industrial Gases (Xiamen, China) for the synthesis, cross-linking, pyrolysis, and sintering of SSP. All reaction processes were carried out using Schlenk techniques [26], under inert atmospheres.

### 2.2. Materials Preparation

#### 2.2.1. Synthesis of Liquid SSPs

Equal molar ratios of HfCl_4_, TaCl_5_, ZrCl_4_, and NbCl_5_ were used as metal sources, with CHCl_3_ as the solvent. The metal chlorides were reacted with AHPCS in weight ratios of 1:2, 1:4, and 1:8, respectively, to prepare a series of liquid SSPs. The obtained liquid SSPs were designated as HTZN-1, HTZN-0.5, and HTZN-0.25, respectively. The ratios of raw materials in the feed used for synthesis of SSPs are shown in Table 1.

Taking the synthesis of HTZN-0.5 as an example, the detailed steps are as follows: Firstly, 0.099 g (0.0004 moL) of ZrCl_4_ and 0.114 g (0.0004 moL) of NbCl_5_ powders were placed into a 100 mL three-necked flask. The flask was purged with nitrogen and sealed using Schlenk techniques. Then, 20 mL of CHCl_3_ was added and stirred evenly. Next, 2.00 g of AHPCS was dissolved in 30 mL of CHCl_3_ to prepare a solution. The solution was transferred into a dropping funnel attached to the flask via a syringe. The dropping rate was adjusted to 20–30 drops/min, and AHPCS solution was added dropwise to the flask at a constant rate. The addition was completed within 0.5 h, and stirring was continued until the solids were dissolved. Then, 0.136 g (0.0004 moL) of HfCl_4_ and 0.152 g (0.0004 moL) of TaCl_5_ were added to the flask. After stirring to obtain a brown solution, the temperature of the solution was raised to 60 °C, and the reaction was kept at this temperature for 1 h. After removal of CHCl_3_ in vacuum distillation, a brown liquid HTZN-0.5 was obtained. For comparison, a blank sample was prepared by AHPCS without metal chlorides under the same conditions (maintained at 60 °C for 1 h), named AHPCS-blank.

#### 2.2.2. Cross-Linking and Pyrolysis of Precursors

The SSP underwent cross-linking at 160 °C for 6 h under a nitrogen flow in a three-necked flask, resulting in the formation of a black solid. The cross-linked SSP was then subjected to pyrolysis in a tube furnace under argon flow at 900 °C for 2 h, producing amorphous ceramic powders. Subsequently, the ceramic powder obtained was annealed in the temperature range of 1100–1600 °C for 2 h and cooled to room temperature, resulting in the SiC/(Hf_0.25_Ta_0.25_Zr_0.25_Nb_0.25_)C/C nanocomposites.

#### 2.2.3. Sintering of SiC/(Hf_0.25_Ta_0.25_Zr_0.25_Nb_0.25_)C/C

**Figure 1:**
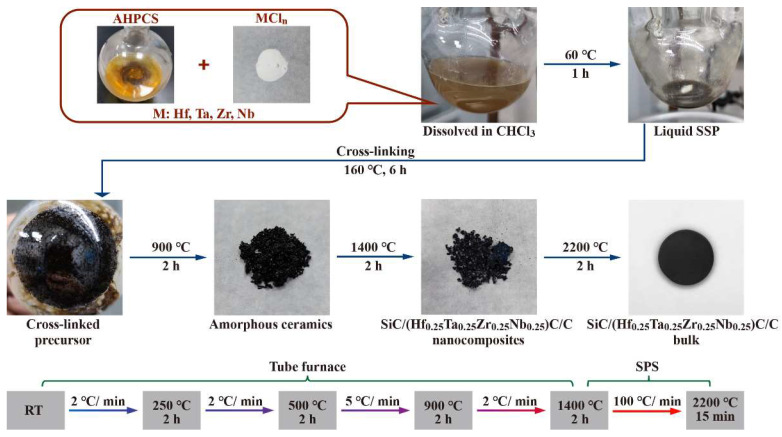
Synthesis of liquid SSP and preparation from precursor to bulk ceramics [27].

The SiC/(Hf_0.25_Ta_0.25_Zr_0.25_Nb_0.25_)C/C powders prepared at 1400 °C were sintered using SPS technology. The sintering process was conducted under an argon atmosphere at a pressure of 50 MPa, with a sintering temperature of 2200 °C and a holding time of 15 min. This SPS process resulted in the formation of monolithic SiC/(Hf_0.25_Ta_0.25_Zr_0.25_Nb_0.25_)C/C nanocomposite, named HTZN-0.5-bulk. The synthesis process from liquid SSP to monolithic ceramic and the thermal treatment steps are illustrated in Figure 1.

### 2.3. Characterization

The molecular structure of the synthesized SSPs was characterized using a Fourier-transform infrared spectrometer (FT-IR, Nicolet iS10, Thermo Fisher Scientific, Waltham, MA, USA). A thermal behavior analysis of the SSPs was conducted using a thermogravimetric analyzer (TGA, STA 449 F5 Jupiter, NETZSCH, Selb, Gemany). The phase composition of ceramic powders was analyzed using an X-ray diffractometer (XRD, SmartLab-SE, Rigaku Corporation, Tokyo, Japan), with Cu Kα1 radiation (λ = 1.5406 Å) as the radiation source. The microstructure and phase composition of the samples were analyzed using a transmission electron microscope (TEM, Talos F200S, FEI, Hillsboro, OR, USA) and selected-area electron diffraction (SAED) techniques. The HTZN-0.5 ceramic powders were sintered to prepare ceramic monoliths using a spark-plasma-sintering furnace (SPS, CXSPS-40A, Chenxin Corporation, Dongguan, China) at 2200 °C for 15 min. The surface morphology of the HTZN-0.5-bulk was observed using a field emission scan electron microscope (SEM, CLARA, TESCAN, Brno, Czech). Hardness and elastic modulus of the HTZN-0.5-bulk were measured using a nanoindenter (UNHT^3^, Anton Paar, Shanghai, China) equipped with a Berkovich indenter and a maximum load of 25 mN. Before the test began, a rectangular area was selected, and three points were chosen along its diagonal. The specimen’s modulus were computed using an Oliver–Pharr method from the reduced modulus [28]. Three evenly spaced indentation tests were conducted, and the average of the highest and lowest values was taken for comparison with previous work. The fracture toughness of the HTZN-0.5-bulk was also measured via the nanoindentation method using a Vickers diamond indenter (MCT^3^, Anton Paar, China) with a maximum load of 5 N. A total of 5 data points were collected. Since the crack-to-indent ratios were as low as between 1.7 and 2.0, a Niihara equation was used to calculate fracture toughness [29]; the result was the average of the highest and lowest values. The volume density and open porosity of the HTZN-0.5-bulk were measured using the water immersion method.

## 3. Results and Discussion

### 3.1. Precursor Characterization

**Figure 2:**
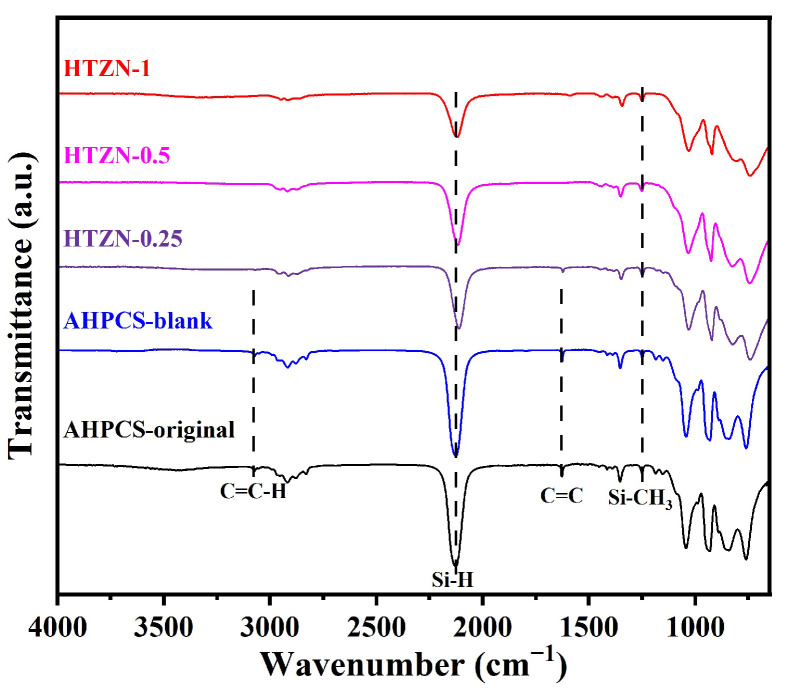
FT-IR spectra of AHPCS, AHPCS-blank, and SSPs.

FT-IR analysis was conducted on the SSPs, and the results are shown in Figure 2. The infrared absorption peaks of AHPCS are consistent with literature reports [30]. In general, both the hydrosilylation (between C=C and Si–H) and dehydrocoupling (between Si–H) in the AHPCS can consume Si–H groups [31]. However, for the AHPCS-blank, the intensity of the absorption peak of C=C–H at 3077 cm^−1^ and the absorption peak of Si–H at 2130 cm^−1^ show nearly no changes compared to the sample AHPCS-original, indicating that neither hydrosilylation nor dehydrocoupling starts at 60 °C.

Our previous studies confirmed that MCl_n_ (M = Hf, Ta, Zr, Nb) can be incorporated into the AHPCS through a dehydrochlorination reaction (between M–Cl and Si–H) [26,32,33]. In the present study, it is assumed that the dehydrochlorination between MCl_n_ (M = Hf, Ta, Zr, Nb) and the AHPCS results in the consumption of Si–H, introducing Hf, Ta, Zr, and Nb into the AHPCS chains, leading to a decrease in the intensity of the Si–H infrared absorption peak, as shown in Figure 2. Furthermore, the disappearance of the absorption peaks of C=C–H and C=C in SSPs suggests that the addition of transition metal chlorides promotes the occurrence of the hydrosilylation (between C=C and Si–H).

The non-reactive Si–CH_3_ peak was used as reference to compare the reaction degree of Si–H in the SSPs with different MCl_n_ (M = Hf, Ta, Zr, Nb) feeding ratio. The semi-quantitative calculation of the Si–H reaction degree $P_{Si-H}$ in SSPs was performed using the formula shown in Equation (1). The results are presented in Table 2.

(1) $$P_{Si-H}=\frac{{\left(A_{Si-H}/A_{Si-{CH}_{3}}\right)}_{AHPCS}-{\left(A_{Si-H}/A_{Si-{CH}_{3}}\right)}_{precursors}}{{\left(A_{Si-H}/A_{Si-{CH}_{3}}\right)}_{AHPCS}}$$

As the MCl_n_ (M = Hf, Ta, Zr, Nb) feed ratio increases, the Si–H reaction extent in the liquid SSPs significantly increases, indicating that more MCl_n_ in the feed consumed more Si–H groups.

Additionally, the Si–H reaction degree in the high-entropy ceramic precursors with the same MCl_n_ (M = Hf, Ta, Zr, Nb) feed ratio was compared to that of the previously reported SiC/HfC/C and SiC/(Hf_0.2_Ta_0.8_)C/C ceramic precursors. According to the Lewis acid-base theory, the compounds in MCl_n_ (M = Hf, Ta, Zr, Nb) act as neutral Lewis acids, with their acidity influenced by the central atom’s radius and valence state. The electronegativities of Zr, Nb, Hf, and Ta are 1.33, 1.60, 1.30, and 1.50, respectively, whereas chlorine has a much higher electronegativity of 3.16, causing the electron density in M–Cl bonds to shift towards the Cl atom. Among the MCl_n_ compounds, ZrCl_4_ exhibits the lowest reactivity due to Zr’s larger atomic radius and lower maximum valence state. Furthermore, before SSP pyrolysis, the dehydrochlorination reaction continues with increasing temperature. Consequently, under the same synthesis conditions, the less reactive ZrCl_4_ consumes less Si–H, resulting in a lower Si–H reaction degree in the SSPs of high-entropy ceramics.

**Figure 3:**
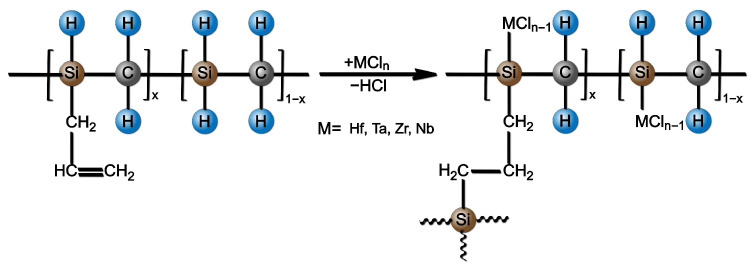
Ideal reaction pathway during the synthesis of liquid SSPs from AHPCS and metal chlorides [27].

According to the FT-IR results, the ideal reaction pathway of liquid SSPs is illustrated in Figure 3. The main chemical reactions include the following: (1) dehydrochlorination between metal chlorides and AHPCS; (2) hydrosilylation of AHPCS.

### 3.2. Cross-Linking and Ceramization of SSPs

**Figure 4:**
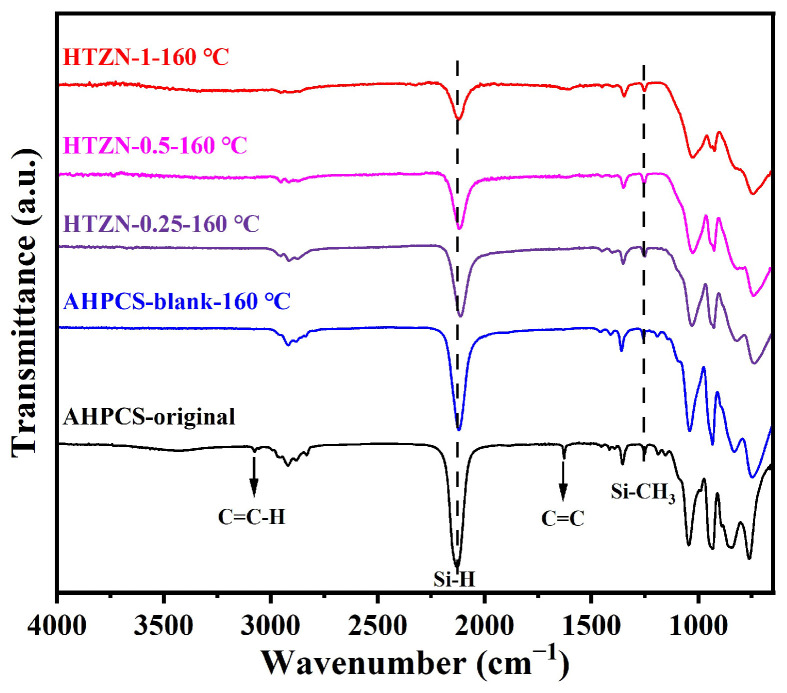
FT-IR spectra of AHPCS, AHPCS-blank, and SSPs cross-linked at 160 °C for 6 h.

The FT-IR spectra of the precursors after crosslinking at 160 °C are shown in Figure 4. Compared to the cross-linked blank sample, AHPCS-blank-160 °C, all the cross-linked SSPs show a significant decrease in the intensity of the Si–H absorption peak at 2130 cm^−1^, confirming that the cross-linking of SSPs is achieved by further consuming Si–H through the dehydrochlorination (M–Cl/Si–H) reaction.

**Figure 5:**
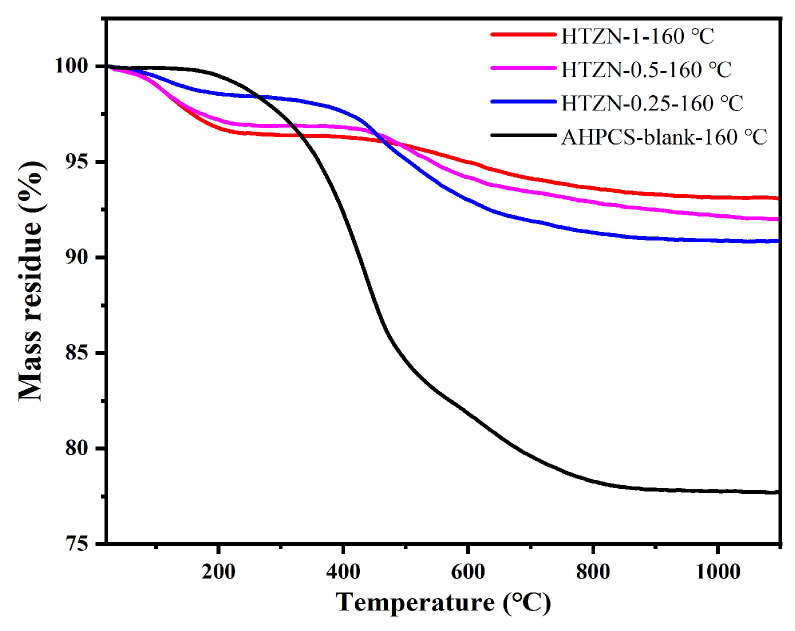
TGA curves of cross-linked SSPs and AHPCS-blank at 160 °C.

The TGA analysis of the cross-linked precursors is presented in Figure 5. The weight loss of AHPCS-blank begins at 200 °C, attributed to the release of hydrogen gas from the dehydrocoupling (occurring between Si–H bonds). In contrast, due to the further dehydrochlorination, the weight loss of SSPs initiates at room temperature, with a mass loss of approximately 1.8 wt.% at 200 °C, slightly higher than that of AHPCS-blank. In the temperature range of 200–900 °C, the mass losses of the cross-linked HTZN-1, HTZN-0.5, HTZN-0.25, and AHPCS-blank are 96.42%, 95.14%, 92.33%, 78.04%, respectively, indicating that the introduction of transition metal chlorides into the AHPCS improves the cross-linking at 160 °C, thereby reducing the mass loss during the ceramization process. The ceramic yields of cross-liked HTZN-1, HTZN-0.5, HTZN-0.25, and AHPCS-blank at 900 °C are 93.3%, 92.5%, 91.0%, and 77.9%, respectively. The SSPs exhibit only slight mass loss between 900 °C and 1100 °C, indicating that the ceramic transformation of SSPs is essentially completed at around 900 °C. Compared to the AHPCS-blank, the SSPs possess higher ceramic yields due to the introduction of transition metal chlorides, which promote the cross-linking of AHPCS [34]. At 1100 °C, the ceramic yields of HTZN-1, HTZN-0.5, and HTZN-0.25 are 93.1%, 92.0%, and 90.9%, respectively, significantly higher than AHPCS-blank.

**Figure 6:**
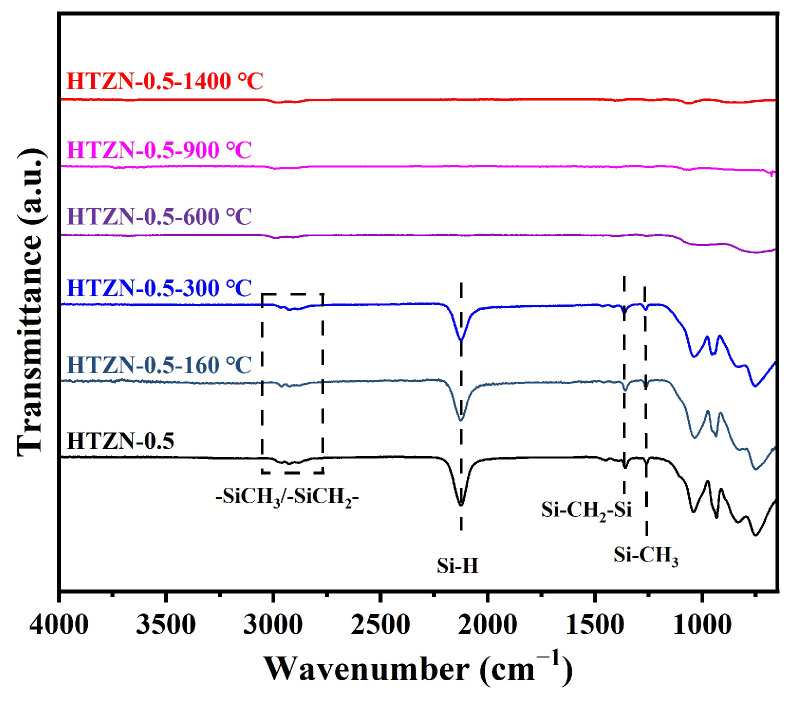
FT-IR spectra of HTZN-0.5 treated at different temperatures.

To investigate the structural evolution of SSPs during the polymer-to-ceramic transformation process, taking the precursor HTZN-0.5 as an example, FT-IR analysis was again conducted on SSPs treated at different temperatures, and the results are shown in Figure 6. As the temperature increases from 160 °C to 600 °C, the intensity of Si–H gradually decreases due to dehydrochlorination and dehydrocoupling reactions, until the Si–H absorption peak completely disappears at 600 °C. Additionally, the intensities of the Si–CH_2_–Si and Si–CH_3_ absorption peaks at 1356 cm^−1^ and 1253 cm^−1^ significantly decrease, attributed to the decomposition of organic groups in SSPs during the ceramic transformation at high temperatures [35,36,37], corresponding to the mass loss observed in Figure 5. At 900 °C, the absorption peak of organic groups has completely disappeared, indicating that the ceramic transformation was completed, which is consistent with the results of the TGA. At 1400 °C, a broad absorption peak appears at 750 cm^−1^, attributed to the amorphous SiC structure [38].

### 3.3. Phase Composition and Microstructural Evolution of Ceramics

**Figure 7:**
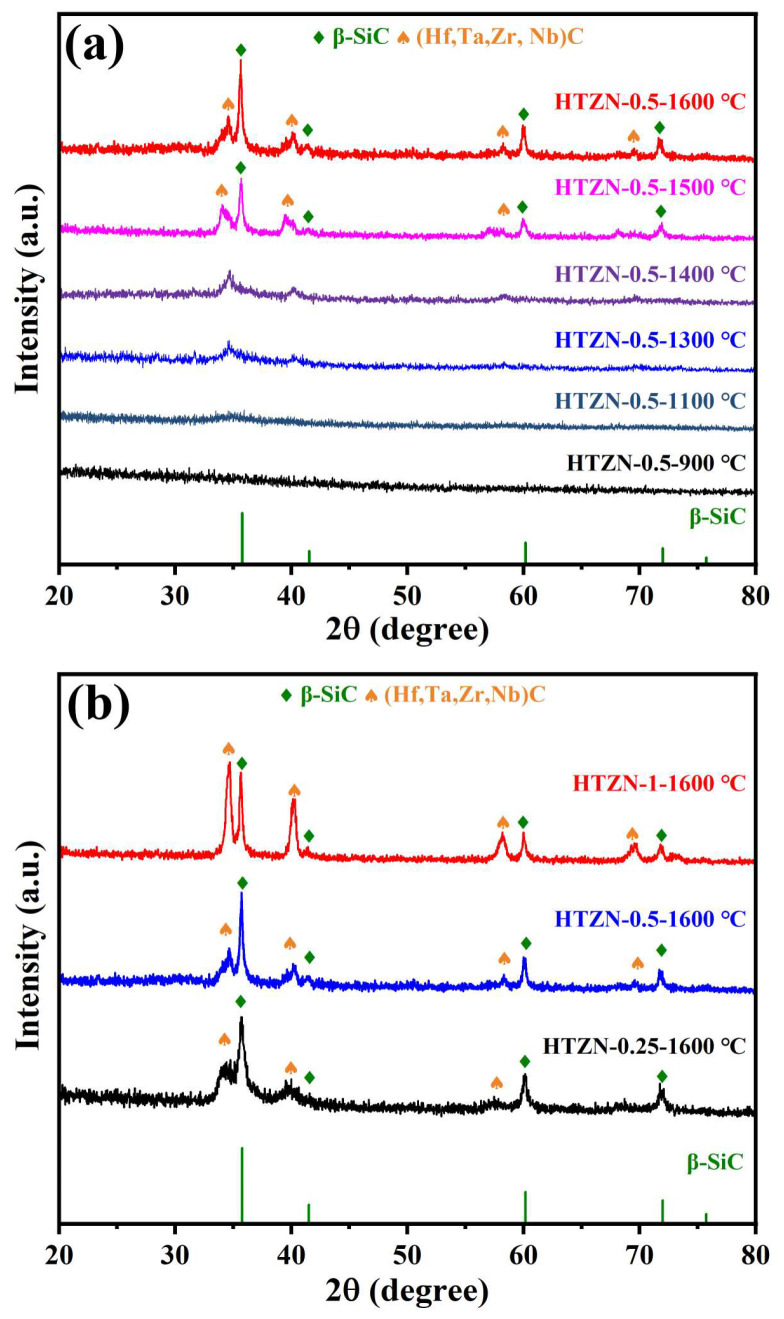
XRD patterns of (**a**) HTZN-0.5-derived ceramics annealed at different temperatures and (**b**) HTZN-0.25-, HTZN-0.5-, HTZN-1-derived ceramics annealed at 1600 °C.

XRD was utilized to analyze the phase composition and microstructural evolution of ceramic powders annealed at different temperatures, and the results are shown in Figure 7a. At 900 and 1100 °C, no obvious diffraction peaks are observed, indicating its amorphous state. At 1300 °C, diffraction peaks attributed to (Hf_0.25_Ta_0.25_Zr_0.25_Nb_0.25_)C emerge. The characteristic diffraction peak of SiC (JCPDS: 75-0254) does not appear in the XRD pattern until 1500 °C. Additionally, the diffraction angle of (Hf_0.25_Ta_0.25_Zr_0.25_Nb_0.25_)C crystal planes appears between HfC (JCPDS: 73-0475) and NbC (JCPDS: 38-1364), gradually shifting towards lower angles in the annealing temperature range from 1300 °C to 1600 °C. This shift is attributed to the alteration of lattice constants in high-entropy phases, which are gradually formed by Hf, Ta, Zr, Nb, and C as the annealing temperature increases. The XRD patterns of samples after annealing at 1600 °C, prepared with different MCl_n_/AHPCS feed ratios, are shown in Figure 7b. The increase in the intensity of diffraction peaks corresponding to high-entropy phases with the increasing transition metal content indicates that by altering the feed ratios during synthesis, molecular design of SSPs can be employed to control the phase composition of SiC/(Hf_0.25_Ta_0.25_Zr_0.25_Nb_0.25_)C/C composite ceramics.

**Figure 8:**
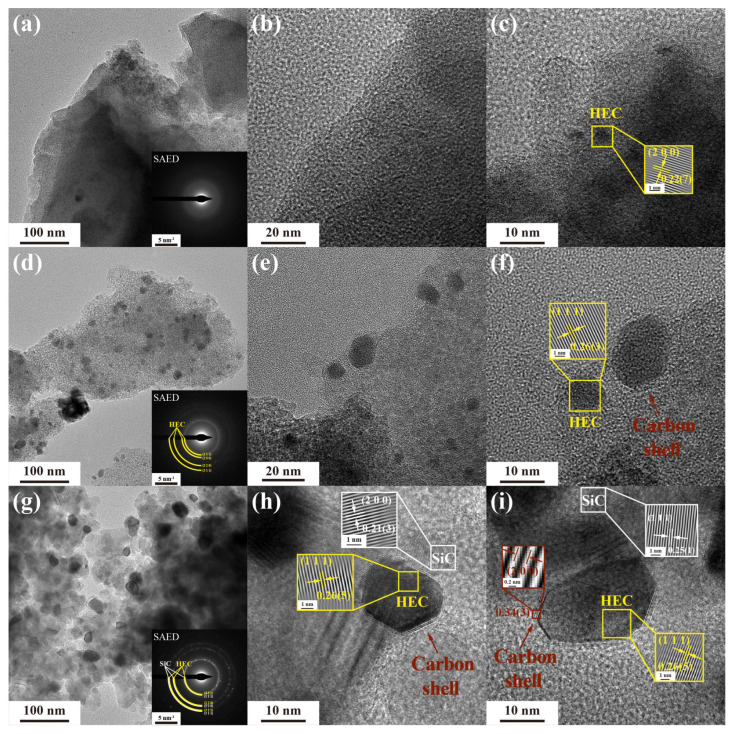
TEM images of (**a**–**c**) HTZN-0.5-900 °C ceramics, (**d**–**f**) HTZN-0.5-1300 °C ceramics, and (**g**–**i**) HTZN-0.5-1600 °C. Insets in images (**a**,**d**,**g**) are selected area electron diffraction (SAED), and insets in images (**c**,**f**,**h**,**i**) are diffraction patterns obtained by inverse Fourier transform (IFFT).

The TEM analysis was conducted to investigate the microstructural evolution of ceramic powders annealed at different temperatures. The selected area electron diffraction (SAED) pattern in Figure 8a does not reveal clear diffraction rings or spots, indicating predominantly amorphous structure of the ceramics after annealing at 900 °C. Figure 8b shows the amorphous feature. The high-resolution transmission electron microscopy (HRTEM) in Figure 8c reveals lattice fringes with a width of 0.22(7) nm, corresponding to the (2 0 0) lattice planes of (Hf_0.25_Ta_0.25_Zr_0.25_Nb_0.25_)C, indicating that the phase segregation of (Hf_0.25_Ta_0.25_Zr_0.25_Nb_0.25_)C starts at 900 °C. At 1300 °C, the SAED pattern in Figure 8d exhibits characteristic polycrystalline diffraction rings of (Hf_0.25_Ta_0.25_Zr_0.25_Nb_0.25_)C, consistent with the XRD results. In addition, the (Hf_0.25_Ta_0.25_Zr_0.25_Nb_0.25_)C nanocrystals are homogenously distributed in the ceramic matrix, with sizes below 20 nm, as shown in Figure 8e. Furthermore, lattice fringes with a width of 0.26(3) nm, corresponding to the lattice planes (1 1 1) of (Hf_0.25_Ta_0.25_Zr_0.25_Nb_0.25_)C, are observed in Figure 8f. In general, the grain size of (Hf_0.25_Ta_0.25_Zr_0.25_Nb_0.25_)C ranges from 5 to 20 nm from Figure 8d–f. Additionally, in situ-formed core–shell structures of (Hf_0.25_Ta_0.25_Zr_0.25_Nb_0.25_)C@C are observed in Figure 8f. Upon increasing the temperature to 1600 °C, formation of in situ SiC@C core–shell structures within the ceramic matrix is observed, with increased ordering of the carbon shell compared to that at 1300 °C. The grain size increases with the annealing temperature increasing, ranging from 50 to 100 nm at 1600 °C, as shown in Figure 8g. The core–shell structure, where the ceramic grains are encapsulated by carbon shells, is expected to inhibit grain growth at high temperatures, thereby improving the high temperature resistance and mechanical properties of the ceramics [23]. In Figure 8h, lattice fringes with widths of 0.26(5) nm and 0.21(3) nm are observed, corresponding to the lattice planes (2 0 0) of (Hf_0.25_Ta_0.25_Zr_0.25_Nb_0.25_)C and (1 1 1) of β-SiC, respectively. Additionally, in Figure 8i, lattice fringes with a width of 0.34(3) nm are observed, indicating that the carbon shell encapsulating the grains has undergone significant graphitization. It is well known that high-entropy ceramics of transition metal carbides exhibit a single-phase cubic rock-salt structure [39]. The relationship between its lattice constants and the composition conforms to Vegard’s law [40,41,42,43], The standard lattice constants for HfC, TaC, ZrC, and NbC are 4.645 Å, 4.454 Å, 4.693 Å, and 4.470 Å [44,45,46,47], respectively. Theoretically, the calculated lattice constant for (Hf_0.25_Ta_0.25_Zr_0.25_Nb_0.25_)C is 4.565 Å. According to the crystal plane spacing of 0.26(5) nm and its corresponding Miller indices (1 1 1) in Figure 8h,i, the lattice constant *a* of (Hf_0.25_Ta_0.25_Zr_0.25_Nb_0.25_)C was calculated using the orthorhombic crystal system lattice constant relationship shown in Equation (2). The calculated value of lattice constant *a* for (Hf_0.25_Ta_0.25_Zr_0.25_Nb_0.25_)C was found to be 0.45(9) nm, which is close to the theoretical value of 4.565 Å predicted by Vegard’s law:

(2) $$d=\frac{a}{\sqrt{\left(h^{2}+k^{2}+l^{2}\right)}}$$

in which, *d* represents the interplanar spacing, *a* represents the lattice constant, and (*h k l*) represents the Miller indices of the corresponding crystal plan.

**Figure 9:**
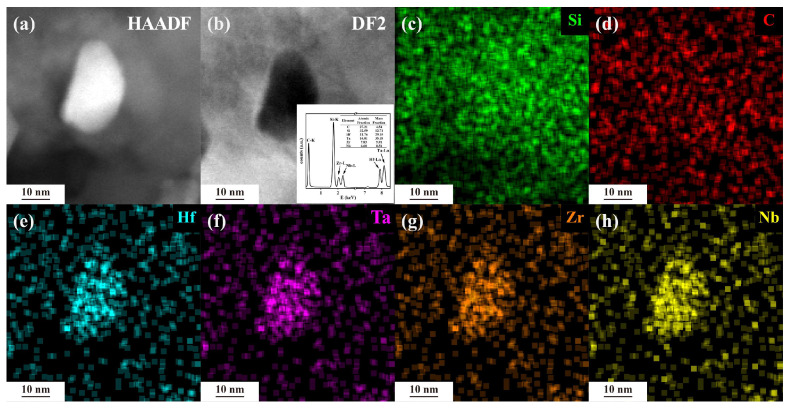
TEM images of HTZN-0.5-1600 °C ceramics: (**a**) high-resolution bright-field image, (**b**) high-angle annular dark-field image (HAADF). (**c**–**h**) The EDS spectrum shows the elemental distribution of Si, C, Hf, Ta, Zr, and Nb, in the SiC/(Hf_0.25_Ta_0.25_Zr_0.25_Nb_0.25_)C/C nanoparticles.

Additionally, elemental mapping was conducted on the HTZN-0.5-1600 °C sample (Figure 9), revealing a uniform distribution of Hf, Ta, Zr, and Nb elements within the (Hf_0.25_Ta_0.25_Zr_0.25_Nb_0.25_)C @C nanoparticles. The elemental characteristic X-ray spectrum in Figure 9b demonstrates the approximate equimolar presence of Hf, Ta, Zr, and Nb, further confirming the in situ formation of high-entropy phases within the matrix.

### 3.4. Phase Composition and Mechanical Properties of Ceramic Monolith

**Figure 10:**
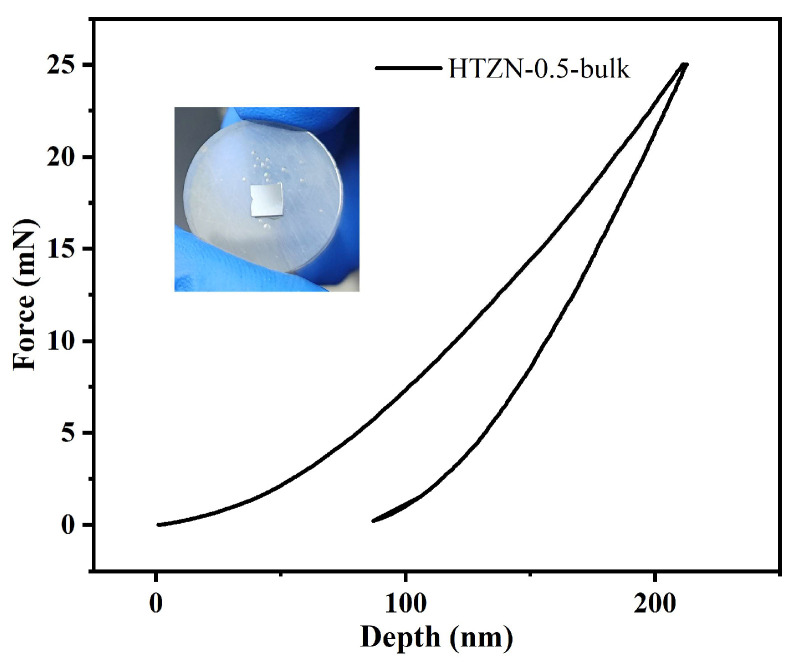
Load–displacement curve of HTZN-0.5-bulk.

Using powdered HTZN-0.5-1400 °C ceramics as the raw materials, a dense monolithic sample, HTZN-0.5-bulk, was prepared using the spark-plasma-sintering (SPS) technique, and its volume density and open porosity were tested (Table 3). The results indicate that the volume density of HTZN-0.5-bulk is approximately 3.34 g/cm^3^, with an open porosity of about 0.41 vol%. The mechanical properties of HTZN-0.5-bulk were tested using a nanoindenter, and the load–displacement curve is shown in Figure 10. The ceramic surface image in Figure 10 shows that the ceramic surface was polished to a mirror-like finish prior to testing in order to avoid surface irregularities affecting the test results. The HTZN-0.5-bulk material exhibits a nano-hardness of 27.47 ± 0.46 GPa, an elastic modulus of 324.00 ± 13.60 GPa, and a fracture toughness of 3.59 ± 0.24 MPa·m^0.5^. As shown in Table 4, due to solid solution strengthening caused by lattice distortion, the mechanical properties of the monolithic HTZN-0.5 material have been improved by nearly four times compared to the monolithic SiC/(Hf_0.2_Ta_0.8_)C/C material prepared under the same conditions [27].

**Figure 11:**
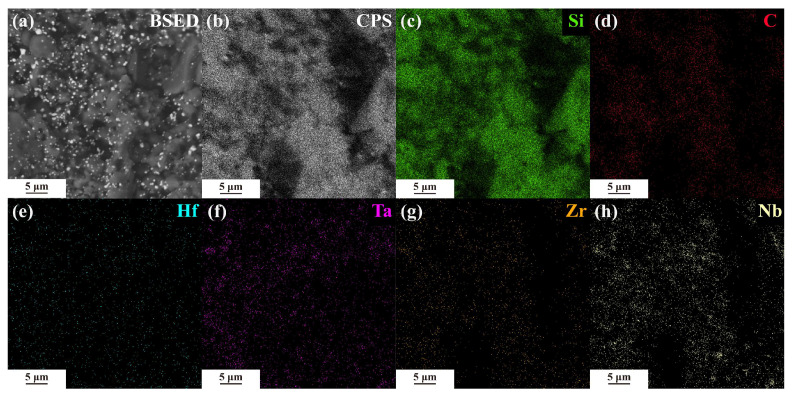
The SEM images of HTZN-0.5-bulk: (**a**) Back-scattered electron image (BSED). (**b**) The distribution of counts per second (CPS) in the energy-dispersive X-ray spectrum. (**c**–**h**) The EDS spectra show the elemental distribution of Si, C, Hf, Ta, Zr, and Nb in the SiC/(Hf_0.25_Ta_0.25_Zr_0.25_Nb_0.25_)C/C bulk sample.

**Figure 12:**
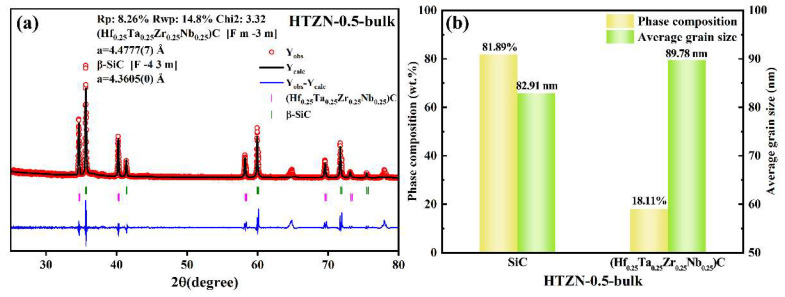
(**a**) The XRD pattern and Rietveld refinement results for the SPS sample HTZN-0.5-bulk. Y_obs_ and Y_calc_ represent the experimental pattern (in red) and calculated pattern (in black), with the blue line at the bottom indicating the fitting line. (**b**) The composition of crystalline phases and their grain sizes in HTZN-0.5-bulk.

The SEM image of HTZN-0.5-bulk is shown in Figure 11. Figure 11a presents the BESD image of HTZN-0.5-bulk, illustrating the dispersion of (Hf_0.25_Ta_0.25_Zr_0.25_Nb_0.25_)C in SiC. Figure 11b shows the distribution of counts per second in the energy-dispersive spectrum, while Figure 11c–h indicate a uniform distribution of Hf, Ta, Zr, Nb, Si, and C. Further refinement of the crystal structure of (Hf_0.25_Ta_0.25_Zr_0.25_Nb_0.25_)C was performed using the Fullprof software for Rietveld refinement of the XRD spectrum. The peak shapes were fitted using the pseudo-Voigt function, and the refined results are shown in Figure 12a. The obtained profile residual factor (*Rp*) is 8.26%, weighted profile residual factor (*Rwp*) is 14.8%, and goodness of fit (*Chi 2*) is 3.32, indicating a good fit with experimental raw data. The lattice constants of SiC and (Hf_0.25_Ta_0.25_Zr_0.25_Nb_0.25_)C are listed in Figure 12a, with the lattice constant of (Hf_0.25_Ta_0.25_Zr_0.25_Nb_0.25_)C being 4.478 Å, showing a negative deviation relative to the theoretical value predicted by Vegard’s law. Figure 12b compares the crystalline phase composition and grain size of HTZN-0.5-bulk, showing that the mass fractions of β-SiC and (Hf_0.25_Ta_0.25_Zr_0.25_Nb_0.25_)C are 81.89% and 18.11%, respectively. The average grain size of both phases is less than 90 nm, confirming that HTZN-0.5-bulk is a nanocomposite ceramic.

## 4. Conclusions

Using AHPCS, HfCl_4_, TaCl_5_, ZrCl_4_, and NbCl_5_ as raw materials, a liquid SSP for high-entropy carbides nanocomposites was prepared. After crosslinking, the ceramic yields of the obtained SSPs exceed 90% at 1100 °C, which paves the way for the further preparation of dense monolithic nanocomposites. By adjusting the feed ratio, the composition of the SiC/(Hf_0.25_Ta_0.25_Zr_0.25_Nb_0.25_)C/C nanocomposite was controlled. Combining PDC and SPS techniques, a dense monolithic SiC/(Hf_0.25_Ta_0.25_Zr_0.25_Nb_0.25_)C/C nanocomposite with an open porosity of 0.41 vol% was successfully fabricated. The mass fractions of β-SiC and (Hf_0.25_Ta_0.25_Zr_0.25_Nb_0.25_)C crystalline phases are 81.89% and 18.11%, respectively, with the average grain sizes of both phases being less than 90 nm. The HTZN-0.5-derived bulk material exhibits a nano-hardness of 27.47 ± 0.46 GPa, an elastic modulus of 324.00 ± 13.60 GPa, and a fracture toughness of 3.59 ± 0.24 MPa·m^0.5^, showing significantly improved mechanical properties compared to the previously reported SiC/(Hf_0.2_Ta_0.8_)C/C bulk material. The liquid SSPs in the present study are key materials to produce the ceramic matrix of continuous fiber-reinforced ceramic matrix composites via the polymer infiltration pyrolysis method. The resultant bulk SiC/(Hf_0.25_Ta_0.25_Zr_0.25_Nb_0.25_)C/C nanocomposite, with its excellent mechanical properties, is a promising candidate for high temperature thermal protection components in aerospace applications.

## Figures and Tables

**Table 1:** Ratios of raw materials in the feed used for synthesis of SSPs.

Liquid SSPs	HfCl_4_: TaCl_5_: ZrCl_4_: NbCl_5_ Molar Ratio	MCl_n_: AHPCS Weight Ratio
HTZN-1	1: 1: 1: 1	1:2
HTZN-0.5	1: 1: 1: 1	1:4
HTZN-0.25	1: 1: 1: 1	1:8

**Table 2:** Reaction degree of Si–H in AHPCS, AHPCS-blank, and SSPs.

Sample	$A_{Si-H}$	$A_{Si-{CH}_{3}}$	$P_{Si-H}$
LAH-0.5 ^a^	71.065	4.053	85.97%
LAH2T8 ^b^	55.913	1.867	76.03%
HTZN-1	13.052	0.360	70.98%
HTZN-0.5	22.980	0.506	63.65%
HTZN-0.25	27.798	0.564	60.56%
AHPCS-blank	45.803	0.380	3.54%
AHPCS	47.732	0.382	-

^a^: The precursor of SiC/HfC/C nanocomposites with the same weight ratio of raw materials in the feed as HTZN-0.5 [24]. ^b^: The precursor of SiC/(Hf_0.2_Ta_0.8_)C/C nanocomposites with the same weight ratio of raw materials in the feed as HTZN-0.5 [27].

**Table 3:** Open porosity and apparent density of HTZN-0.5-bulk sample.

Sample	Dry Weight (mg)	Wet Weight (mg)	Buoyant Weight (mg)	Open Porosity	Bulk Density (g/cm^3^)
HTZN-0.5-bulk	89.09	89.20	62.55	0.41%	3.34

**Table 4:** Hardness and elastic modulus of HTZN-0.5-bulk.

Sample	HIT (GPa)	EIT (GPa)	K_IC_ (MPa·m^0.5^)
LAH2T8-bulk ^a^	7.72 ± 1.36	76.71 ± 7.89	-
HTZN-0.5-bulk	27.47 ± 0.46	324.00 ± 13.60	3.59 ± 0.24

^a^: Monolithic SiC/(Hf_0.2_Ta_0.8_)C/C nanocomposites [27].

## Data Availability

The data presented in this study are available on request from the corresponding author.
